# Supplementary material for: Prevalence and factors associated with physical function limitation in older West African people living with HIV
Source: PLoS One. 2020 Oct 22;15(10):e0240906. doi: 10.1371/journal.pone.0240906 (PMC7580884; doi:10.1371/journal.pone.0240906)
Supplement: S1 Fig — (DOCX) [file pone.0240906.s001.docx]

**S1 Fig. Distribution of SPPB scores in the study population***

* Scores range from 0 to 12. According to Guralnik et al, 2000, a score from 7 to 9 is considered as moderately impaired and a score ≤6 is considered as severely impaired.

Reference :

Guralnik JM, Ferrucci L, Pieper CF, Leveille SG, Markides KS, Ostir GV, et al. « Lower extremity function and subsequent disability: consistency across studies, predictive models, and value of gait speed alone compared with the short physical performance battery » [J. Gerontol. A Biol. Sci. Med. Sci. 2000 avr;55(4):M221-231.](http://www.ncbi.nlm.nih.gov/pubmed/10811152)
