# Supplementary material for: Prevalence and factors associated with physical function limitation in older West African people living with HIV
Source: PLoS One. 2020 Oct 22;15(10):e0240906. doi: 10.1371/journal.pone.0240906 (PMC7580884; doi:10.1371/journal.pone.0240906)
Supplement: S2 Fig — (DOCX) [file pone.0240906.s002.docx]

**S2 Fig. Distribution of scores for each SPPB subtests in the study population***

* For each SPPB subtest (ie balance, Gait speed and 5 Sit-To-Stand), scores range from 0 to 4
