# Supplementary material for: Prevalence and factors associated with physical function limitation in older West African people living with HIV
Source: PLoS One. 2020 Oct 22;15(10):e0240906. doi: 10.1371/journal.pone.0240906 (PMC7580884; doi:10.1371/journal.pone.0240906)
Supplement: S1 Table — (DOCX) [file pone.0240906.s003.docx]

| Difficulties with : | No | Mild | Moderate | Severe | Extreme /  Cannot do it |
| --- | --- | --- | --- | --- | --- |
| - climbing stairs without help | 231 (69.4%) | 55 (16.5%) | 40 (12.0%) | 7 (2.1%) | 0 (0.0%) |
| - raising arm | 311 (93.4%) | 13 (3.9%) | 8 (2.4%) | 1 (0.3%) | 0 (0.0%) |
| - carrying a 5 kg grocery bag over a distance of 10 meters without help* | 285 (85.6%) | 19 (5.7%) | 20 (6.0%) | 4 (1.2%) | 4 (1.2%) |
| - walking 1 kilometer * | 295 (88.6%) | 24 (7.2%) | 12 (3.6%) | 0 (0.0%) | 1 (0.3%) |
| - participating in community activities, such as religious services, social activities or voluntary work because of your health | 319 (95.8%) | 7 (2.1%) | 6 (1.8%) | 0 (0.0%) | 1 (0.3%) |
| - visiting relatives or friends because of your health | 319 (95.8%) | 9 (2.7%) | 3 (0.9%) | 2 (0.6%) | 0 (0.0%) |

**S1 Table. Self-reported difficulties in daily physical function**

* 1 missing value
