# Supplementary material for: Prevalence and factors associated with physical function limitation in older West African people living with HIV
Source: PLoS One. 2020 Oct 22;15(10):e0240906. doi: 10.1371/journal.pone.0240906 (PMC7580884; doi:10.1371/journal.pone.0240906)
Supplement: Attachment — Submitted filename: PONE-D-20-01113_reviewer.pdf [file pone.0240906.s004.pdf]

PLOS ONE

Prevalence and factors associated with physical function limitation in older West African people living with HIV

--Manuscript Draft--

**Manuscript Number:**	PONE-D-20-01113
**Article Type:**	Research Article
**Full Title:**	Prevalence and factors associated with physical function limitation in older West African people living with HIV
**Short Title:**	Aging, HIV and physical function in Africa
**Corresponding Author:**	Charlotte Bernard<br>INSERM U1219<br>Bordeaux, FRANCE
**Keywords:**	HIV; aging; physical function; SPPB; Grip strength; Sub-Saharan Africa
**Abstract:**	Although physical function decline is common with aging, the burden of this impairment remains underestimated in patients living with HIV (PLHIV), particularly in the older ones receiving antiretroviral treatment (ART) and living in sub-Saharan Africa (SSA). PLHIV aged ≥50 years old and on ART since ≥6 months were included (N=333) in three clinics (two in Côte d'Ivoire, one in Senegal) participating to the International epidemiological Databases to Evaluate AIDS (IeDEA) West Africa collaboration. Physical function was measured using the Short Physical Performance Battery (SPPB), the unipodal balance test and self-reported questionnaires. Grip strength was also assessed. Logistic regression was used to identify the factors associated with SPPB performance specifically. Median age was 57 (54-61) years, 57.7% were female and 82.7% had an undetectable viral load. Almost 30% had lower SPPB performance with the 5-sit-to-stand test being the most altered subtest (64%). PLHIV with low SPPB performance also had low performance on the unipodal balance test (54.2%) and significant low mean grip strength (only in men: 35.7±9.1 vs 41.9±12.7 kg). Age ≥60 years, being female, having abdominal obesity and a longer disease duration were associated to lower SPPB performance. As in western countries, physical function limitation is now part of the burden of HIV disease complications of older PLHIV living in West Africa, putting this population at higher risk of falls, fracture, and disability. How to screen those impairments and integrate their management in the standards of care should be investigated.
**Order of Authors:**	Charlotte Bernard
	Hélène FONT
	Zélica DIALLO
	Richard AHONON
	Judicaël Malick TINE
	Franklin ABOUO
	Aristophane TANON
	Eugène MESSOU
	Moussa SEYDI
	François DABIS
	Nathalie de Rekeneire
**Additional Information:**	
**Question**	**Response**
**Financial Disclosure**	no

Powered by Editorial Manager® and ProduXion Manager® from Aries Systems Corporation

Enter a financial disclosure statement that describes the sources of funding for the work included in this submission. Review the submission guidelines for detailed requirements. View published research articles from *PLOS ONE* for specific examples.<br><br>This statement is required for submission and **will appear in the published article** if the submission is accepted. Please make sure it is accurate.<br><br>**Unfunded studies**<br>Enter: *The author(s) received no specific funding for this work.*<br><br>**Funded studies**<br>Enter a statement with the following details:<br>• Initials of the authors who received each award<br>• Grant numbers awarded to each author<br>• The full name of each funder<br>• URL of each funder website<br>• Did the sponsors or funders play any role in the study design, data collection and analysis, decision to publish, or preparation of the manuscript?<br>• **NO** - Include this sentence at the end of your statement: *The funders had no role in study design, data collection and analysis, decision to publish, or preparation of the manuscript.*<br>• **YES** - Specify the role(s) played.<br><br>* typeset	
**Competing Interests**<br><br>Use the instructions below to enter a competing interest statement for this submission. On behalf of all authors, disclose any competing interests that could be perceived to bias this work—acknowledging all financial support and any other relevant financial or non-financial competing interests.<br><br>This statement **will appear in the published article** if the submission is accepted. Please make sure it is accurate. View published research articles from *PLOS ONE* for specific examples.	NO authors have competing interests

**NO authors have competing interests**<br><br>Enter: *The authors have declared that no competing interests exist.*<br><br>**Authors with competing interests**<br><br>Enter competing interest details beginning with this statement:<br><br>*I have read the journal's policy and the authors of this manuscript have the following competing interests: [insert competing interests here]*<br><br>* typeset	
**Ethics Statement**<br><br>Enter an ethics statement for this submission. This statement is required if the study involved:<br><br>• Human participants<br>• Human specimens or tissue<br>• Vertebrate animals or cephalopods<br>• Vertebrate embryos or tissues<br>• Field research<br><br>Write "N/A" if the submission does not require an ethics statement.<br><br>General guidance is provided below. Consult the submission guidelines for detailed instructions. **Make sure that all information entered here is included in the Methods section of the manuscript.**	Done

**Format for specific study types**<br><br>**Human Subject Research (involving human participants and/or tissue)**<br>• Give the name of the institutional review board or ethics committee that approved the study<br>• Include the approval number and/or a statement indicating approval of this research<br>• Indicate the form of consent obtained (written/oral) or the reason that consent was not obtained (e.g. the data were analyzed anonymously)<br><br>**Animal Research (involving vertebrate animals, embryos or tissues)**<br>• Provide the name of the Institutional Animal Care and Use Committee (IACUC) or other relevant ethics board that reviewed the study protocol, and indicate whether they approved this research or granted a formal waiver of ethical approval<br>• Include an approval number if one was obtained<br>• If the study involved *non-human primates*, add *additional details* about animal welfare and steps taken to ameliorate suffering<br>• If anesthesia, euthanasia, or any kind of animal sacrifice is part of the study, include briefly which substances and/or methods were applied<br><br>**Field Research**<br><br>Include the following details if this study involves the collection of plant, animal, or other materials from a natural setting:<br>• Field permit number<br>• Name of the institution or relevant body that granted permission	
**Data Availability**<br><br>Authors are required to make all data underlying the findings described fully available, without restriction, and from the time of publication. PLOS allows rare exceptions to address legal and ethical concerns. See the PLOS Data Policy and FAQ for detailed information.	No - some restrictions will apply

Powered by Editorial Manager® and ProduXion Manager® from Aries Systems Corporation

A Data Availability Statement describing where the data can be found is required at submission. Your answers to this question constitute the Data Availability Statement and **will be published in the article**, if accepted.<br><br>**Important:** Stating 'data available on request from the author' is not sufficient. If your data are only available upon request, select 'No' for the first question and explain your exceptional situation in the text box.<br><br>Do the authors confirm that all data underlying the findings described in their manuscript are fully available without restriction?	
**Describe where the data may be found in full sentences. If you are copying our sample text, replace any instances of XXX with the appropriate details.**<br><br>• If the data are **held or will be held in a public repository**, include URLs, accession numbers or DOIs. If this information will only be available after acceptance, indicate this by ticking the box below. For example: *All XXX files are available from the XXX database (accession number(s) XXX, XXX.).*<br>• If the data are all contained **within the manuscript and/or Supporting Information files**, enter the following: *All relevant data are within the manuscript and its Supporting Information files.*<br>• If neither of these applies but you are able to provide **details of access elsewhere**, with or without limitations, please do so. For example:<br><br>*Data cannot be shared publicly because of [XXX]. Data are available from the XXX Institutional Data Access / Ethics Committee (contact via XXX) for researchers who meet the criteria for access to confidential data.*<br><br>*The data underlying the results presented in the study are available from (include the name of the third party*	Complete data for this study cannot be posted in a supplemental file or a public repository at this current time because of scientific reasons here explained. This cross-sectional analysis is part of longitudinal study evaluating different aspects of aging with HIV (cognition, physical function, depression and frailty) with a 2-year follow-up which ended in December 2019. Cross-sectional analyses on cognition, depression and frailty and longitudinal analyses on these topics and physical function are currently in progress and thus data could not be posted at this time.

Powered by Editorial Manager® and ProduXion Manager® from Aries Systems Corporation

and contact information or URL).<br>• This text is appropriate if the data are owned by a third party and authors do not have permission to share the data.<br><br>* typeset	
Additional data availability information:	

Click here to download Manuscript Manuscript_02012020.docx

Prevalence and factors associated with physical function limitation in older West African people living with HIV

Running head: Aging, HIV and physical function in Africa

Authors: Charlotte BERNARD[1,2], Hélène FONT[1,2], Zélica DIALLO[3], Richard AHONON[4], Judicaël Malick TINE[5], Franklin ABOUO[3], Aristophane TANON[3], Eugène MESSOU[4], Moussa SEYDI[5], François DABIS[1,2], Nathalie de REKENEIRE[1,2] and the IeDEA West Africa Cohort Collaboration

[1] Univ. Bordeaux, INSERM, Bordeaux Population Health Research Center, UMR 1219, F-33000 Bordeaux, France
[2] Univ. Bordeaux, ISPED, Bordeaux Population Health Research Center, UMR 1219, F-33000 Bordeaux, France
[3] Service de maladies infectieuses et tropicales, CHU Treichville, Abidjan, Côte d'Ivoire
[4] Centre de prise en charge de recherche et de formation (CePReF), Yopougon Attié Hospital, Abidjan, Côte d'Ivoire
[5] Service de maladies infectieuses et tropicales, CRCF, CHNU de Fann, Dakar, Senegal

Corresponding author: Bernard Charlotte, PhD, Equipe Maladies infectieuses dans les pays à ressources limitées-IDLIC, Centre INSERM U1219, ISPED, Université de Bordeaux 146 rue Léo Saignat, 33076 BORDEAUX Cedex (FRANCE).

Tél: 05 57 57 56 65, Fax: 05 56 24 00 81;

Mail: charlotte.bernard@u-bordeaux.fr

Word count: 3,500; **Tables:** 3

Source of funding

Supported by the National Institute of Mental Health (NIMH), National Cancer Institute (NCI), the Eunice Kennedy Shriver National Institute of Child Health & Human Development (NICHD) and the National Institute of Allergy and Infectious Diseases (NIAID) of the U.S. National Institutes of Health (NIH), as part of the International Epidemiologic Databases to Evaluate AIDS (IeDEA) under Award Number U01AI069919. The content is solely the

responsibility of the authors and does not necessarily represent the official views of the National Institutes of Health. The authors have no conflicts of interest to disclose.

ACKNOWLEDGEMENTS

Supported by the National Institute of Mental Health (NIMH), National Cancer Institute (NCI), the Eunice Kennedy Shriver National Institute of Child Health & Human Development (NICHD) and the National Institute of Allergy and Infectious Diseases (NIAID) of the U.S. National Institutes of Health (NIH), as part of the International Epidemiologic Databases to Evaluate AIDS (IeDEA) under Award Number U01AI069919. The content is solely the responsibility of the authors and does not necessarily represent the official views of the National Institutes of Health. The authors have no conflicts of interest to disclose.

The IeDEA West Africa region: Site investigators and cohorts:
Adult cohorts: Marcel Djimon Zannou, CNHU, Cotonou, Benin; Armel Poda, CHU Souro Sanou, Bobo Dioulasso, Burkina Faso; Fred Stephen Sarfo and Komfo Anokeye Teaching Hospital, Kumasi, Ghana; Eugene Messou, ACONDA CePReF, Abidjan, Cote d'Ivoire; Henri Chenal, CIRBA, Abidjan, Cote d'Ivoire; Kla Albert Minga, CNTS, Abidjan, Cote d'Ivoire; Emmanuel Bissagnene, and Aristophane Tanon, CHU Treichville, Cote d'Ivoire; Moussa Seydi, CHU de Fann, Dakar, Senegal; Akessiwe Akouda Patassi, CHU Sylvanus Olympio, Lomé, Togo. Pediatric cohorts: Sikiratou Adouni Koumakpai-Adeothy,_CNHU, Cotonou, Benin; Lorna Awo Renner, Korle Bu Hospital, Accra, Ghana; Sylvie Marie N'Gbeche, ACONDA CePReF, Abidjan, Ivory Coast; Clarisse Amani Bosse, ACONDA_MTCT+, Abidjan, Ivory Coast; Kouadio Kouakou, CIRBA, Abidjan, Cote d'Ivoire; Madeleine Amorissani Folquet, CHU de Cocody, Abidjan, Cote d'Ivoire; François Tanoh Eboua, CHU de Yopougon, Abidjan, Cote d'Ivoire; Fatoumata Dicko Traore, Hopital Gabriel Toure, Bamako, Mali; Elom Takassi, CHU Sylvanus Olympio, Lomé,Togo; Coordinators and data centers: François Dabis, Elise Arrive, Eric Balestre, Renaud Becquet, Charlotte Bernard, Shino Chassagne Arikawa, Alexandra Doring, Antoine Jaquet, Karen Malateste, Elodie Rabourdin, Thierry Tiendrebeogo, ADERA, Isped & INSERM U1219, Bordeaux, France. Sophie Desmonde, Julie Jesson, Valeriane Leroy, Inserm 1027, Toulouse, France. Didier Koumavi Ekouevi, Jean-Claude Azani, Patrick Coffie, Abdoulaye Cissé, Guy Gnepa, Apollinaire Horo, Christian Kouadio, Boris Tchounga, PACCI, CHU Treichville, Abidjan, Côte d'Ivoire.

Abstract (240 words)

Although physical function decline is common with aging, the burden of this impairment remains underestimated in patients living with HIV (PLHIV), particularly in the older ones receiving antiretroviral treatment (ART) and living in sub-Saharan Africa (SSA). PLHIV aged ≥50 years old and on ART since ≥6 months were included (N=333) i[illegible]ree clinics (two in Côte d'Ivoire, one in Senegal) participating [illegible] the International epidemiological Databases to Evaluate AIDS (IeDEA) West Africa collaboration. Physical function was measured using the Short Physical Performance Battery (SPPB), the unipodal balance test and self-reported questionnaires. Grip strength was also assessed. Logistic regression was used to identify the factors associated with SPPB performance specifically. Median age was 57 (54-61) years, 57.7% were female and 82.7% had an undetectable viral load. Almost 30% had lower SPPB performance with the 5-sit-to-stand test being the most altered subtest (64%). PLHIV with low SPPB performance also had low performance on the unipodal balance test (54.2%) and significant low mean grip strength (only in men: 35.7±9.1 vs 41.9±12.7 kg). Age ≥60 years, being female, having abdominal obesity and a longer disease duration were associated [illegible]ower SPPB performance. As in western countries, physical function limitation is now part of the burden of HIV disease complications of older PLHIV living in West Africa, putting this population at higher risk of falls, fracture, and disability. How to screen those impairments and integrate their management in the standards of care should be investigated.

Keywords: HIV, Aging, Physical function, SPPB, Grip strength, Sub-Saharan Africa

INTRODUCTION

According to UNAIDS, 6.7 million people aged 50 and older were living with HIV worldwide in 2017, a phenomenon that increased steadily since 1995 [1]. Sub-Saharan Africa (SSA) represents the region with the largest number of older people living with HIV (PLHIV) (4.1 million) [1]. With massive and rapid access to antiretroviral therapy (ART), the overall improvement in HIV medical care services and the dramatic change in the demographics of the HIV-infected population, HIV infection can now be considered as a chronic disease worldwide. Older patients receiving ART are thus more and more numerous and at an increased risk of developing age-associated non-communicable comorbidities [2,3].

With aging, alteration of physical function (i.e. functional limitation and disability) are more common. Independence and social life are clearly related to physical function in older adults [4]. Functional limitation is a strong predictor of disability, hospitalization, nursing home admission and mortality in aging [5,6]. In the context of HIV infection, this problem has long been neglected although up to 30% of PLHIV in Canada for instance have complaints about their physical function [7]. Recent US data showed that PLHIV are affected by those impairments even at middle age [8]. In France, it has been reported that one of two middle-aged PLHIV had poor lower limb muscle performance [9] and that the performance deteriorated over time which was associated with subsequent falls [10]. In Cote d'Ivoire, one study reported a higher prevalence of physical function limitation (34%) in middle-aged PLHIV with older age and higher BMI being associated with lower performance [11].

As those impairments emerge as an important public health issue, there is a risk of a significant burden on the healthcare systems and human resources especially in SSA. Few data are available in SSA, particularly in PLHIV aged 50 years and above. The aim of our study was to describe the prevalence and identify factors associated with physical function impairment in PLHIV aged 50 years and above living in West Africa.

MATERIALS & METHODS

Study population

This study is a part of an ancillary project within the West Africa network of the International epidemiological Databases to Evaluate AIDS (IeDEA) of the US National Institutes of Health (https://www.iedea.org/regions/west-africa/) [12]. This cross-sectional analysis is part a 2-year longitudinal study evaluating different aspects of aging with HIV (cognition, physical function, depression and frailty) with a follow-up still ongoing. By convenience, the study was conducted in three urban clinics with a large case load of PLHIV in two different countries: the infectious and tropical disease department of the Treichville University Hospital, and the referral public clinic (CePReF) in Yopougon Attié Hospital (Abidjan, Côte d’Ivoire) and the infectious and tropical disease department of the Fann University Hospital (Dakar, Senegal). The inclusion period of the study was between February 2016 to November 2017.

Patients were eligible if they were adults living with type-1 HIV, 50 years old or older and on ART since at least 6 months. We excluded patients having an history of cerebral opportunistic infection, a neurological pathology (history of stroke, Parkinson disease), a current disabling opportunistic infection, a meningitis, a sensitivo-motor paralysis, a psychiatric illness (including psychotropic treatment), a cancer under treatment or a respiratory or cardiac insufficiency.

The protocol has been approved by the national ethic committee of each participating country. All the patients gave their written consent before being included in the study.

Physical function limitation

Physical function limitation was evaluated with different tests of physical performance or by self-reported measures. These tests were conducted by trained staff following standard procedures. Each time was recorded by a digital stop watch.

Physical performance

- The Short Physical Performance Battery (SPPB) [13] that assesses lower extremity function using three timed components in the following order: balance, gait speed, and lower limb strength (5 times sit-to-stand test - 5STS). A score between 0 to 4 was attributed to each subtest, depending on the performance. The sum of the three components comprised the final SPPB score with a possible range from 0 to 12 (12 indicating the highest degree of lower extremity functioning). For physical limitation, as defined by Guralnik et al [13], poor physical performance was defined as a total SPPB score ≤9. To describe in details which SPPB subtest was the most altered, we used these following cutoffs: a score <4 for balance test, a speed ≤0.8m/s for the walking speed test and a score <4 for the 5STS.
- The unipodal balance test [14] which consists in standing on one leg as long as possible (max 30 seconds), with open eyes. The test was repeated two times. The best performance from the 2 tests was used for the analyses. Lower performance at the unipodal balance test was defined based on normative data [14].
- A “physical function impairment” variable was also described including at least more than one test altered, as defined above.

Self-reported measures

Additionally, daily physical function tasks were evaluated based on self-reported difficulties in daily physical function such as climbing stairs, walking long distance, participating to

community activities, visiting family, etc. Each difficulty was evaluated with a Likert scale (no difficulty / mild / moderate / severe / extreme difficulties or cannot do).

Grip strength

As SPPB assesses lower extremity function and in order to have a complete description of muscular function in our PLHIV, the hand grip strength (kg) was measured to evaluate upper extremity function[illegible] The measure was realized in standard way, twice consecutively in the dominant hand using a calibrated Jamar hydraulic hand dynamometer. The maximum value of the 2 trials was used for the analysis. For grip strength, as no normative data obtained from a population with similar characteristics to ours were available, lower grip strength was defined as a performance less to the median obtained in each gender group (male / female).

Measures of functional status

Activities of the daily living (ADL) [15] and Instrumental activities with daily living (IADL) [16] scales were used to evaluate the autonomy of the patients. The ADL and IADL scores range from 0 to 6 and 0 to 4, respectively (0 indicating the lowest degree of autonomy).

Other covariates

The practice of physical activity was evaluated with the WHO Global Physical Activity Questionnaire (GPAQ) [17], collecting information on physical exer[illegible] in three situations: at work, during moving from one place to another and during hobbies. The last question evaluates inactivity (how many hours do you spend [illegible]or lying down during a day (without sleeping). For the present study, we focused on global scores: inactivity (yes/no) and intensity of physical activity (limited to moderate vs high) which were calculated following the GPAQ analysis guide instructions.

Patients' characteristics including information on socio-demographics, anthropometry, medical history, HIV clinical data and also substance use (tobacco, drugs and alcohol with the AUDIT-C) have been collected through basic questions and medical examination. The Body Mass Index (BMI) was calculated by dividing the weight (in kilograms) by the height (in meters squared) and divided in 3 categories: overweight/obesity when BMI ≥25 kg/m², normal when 18 ≤ BMI <25 and low when <18 kg/m². The abdominal obesity was defined by an abdominal circumference ≥80 cm in women and ≥94 in me[illegible]

Concerning medical data, patients were asked if they have ever been diagnosed with some diseases (hypertension, diabetes, hyperlipidemia, migraine, arthrosis, tuberculosis, etc.). The initial clinical stage using Centers for Disease Control and Prevention (CDC) definition was used (A, B or C). Exposure to raltégravir (RAL), zidovudine (AZT), didanosine (ddI), stavudine (D4T), zalcitibine (DDC) in the initial ART treatment and also in the current ART treatment was studied through a categorical variable (yes/no). Adherence to ART was defined as the percentage of tablets the patient declared to have taken over 7 days (in comparison to the prescribed total number of tablets over this period).

Statistical analysis

The characteristics of the study sample were described using median and interquartile range (IQR) for continuous variables, numbers and proportions for categorical variables.

For each test and for the "physical function impairment" variable, the prevalence of low performance was reported.

As SPPB has been used for >20 years to measure physical performance in older adults [13] and as it is a complete brief battery feasible to implement in HIV clinical care [18], the further analyses focused on this test. Associations between lower SPPB performance and other tests

performance was evaluated: with Chi-2 test for the unipodal balance test and with the Student T test in each gender group separately for the mean grip strength.

Factors associated with lower SPPB performance were evaluated with univariable and multivariable logistic regression analyses. The multivariable logistic regression models included all variables associated with the dependent variable with a p-value ≤ 0.2 in univariable analyses. Unbalanced variables (85%/15%) were excluded from the analyses. The final model was obtained with a backward stepwise selection and we considered significant associations at $p < 0.05$. “Inclusion centers” variable was included as a cofounder in each model. The goodness of fit of the final model was evaluated with the Hosmer-Lemeshow test ($p > 0.05$).

A multivariable imputation of missing data was performed with a Random Forest procedure. No significant difference was observed between the two databases (with and without missing data). The database with imputed missing data was used for the logistic regression analyses.

Statistical analyses were computed using R software.

RESULTS

Characteristics of the sample

A total of 333 patients were included in our study. The median (IQR) age was 57 (54-61) years old. Among them, 35.1% were aged 60 years and older, 57.7% were female, and 50.8% had a primary or less level of education. Almost half of them lived in c[illegible]ple (46.3%) and were active (53.1%) (Table 1).

Table 1. Characteristics of the study sample

	N	%
**N**	333	100
**Socio-demographic data**		
Age		
50-59	216	64.86
60 et +	117	35.14
Gender		
Male	141	42.34
Female	192	57.66
Matrimonial situation		
In couple	154	46.25
Alone	179	53.75
Level of education		
Primary or less	169	50.75
Secondary or more	164	49.25
Professional activity		
Active	177	53.15
Non active	156	46.85
**Anthropometric and medical data**		
Overweight/obesity	126	37.95
Abdominal obesity	177	53.31
Hypertension	73	21.99
Hyperlipidemia	12	3.63
Diabetes	20	6.01
B or C hepatitis	9	2.71
History of tuberculosis	72	21.62
Migraine	34	10.21
Arthrosis	52	15.66
Other medical problem	14	4.24
History of trauma	22	6.65
History of neurological disease	47	14.11
**HIV Clinical data**		
Duration of infecti[illegible]		
[7.29,88.4]	111	33.33
(88.4,131]	112	33.63
(131,317]	110	33.03
Clinical disease stages at ART initiation		
A	100	30.40
B	181	55.02
C	48	14.59
Nadir CD4		
<200	203	62.85
≥200	120	37.15
More recent CD4		
<500	162	49.09
≥500	168	50.91
Detectable Viral load	48	17.33
Initial treatment including RAL, AZT, ddI, D4T, DDC	227	68.17
Current treatment including RAL, AZT, ddI, D4T, DDC	89	26.73
Poor Observance	19	5.71

Table 1 (continued)

	N	%
**Substance use**		
Current drinkers	24	7.21
Tobacco use (current/former)	59	17.72
Drug consumption	6	1.81

Abbreviations: ART: antiretroviral, AZT: zidovudine, ddI: didanosine, DDC: zalcitibine, D4T: stavudine, RAL: raltégravir.

Among these patients, 37.9% were overweight or obese, 53.3% had an abdominal obesity, and 21.9% had hypertension. Less than a quarter ever had a tuberculosis (21.6%). Other comorbidities (diabetes, hyperlipidemia, migraine and arthrosis) were less prevalent (6%, 3.6%, 10.2% and 15.7%, respectively).

A large majority of patients had an undetectable viral load (82.7%), half of them had CD4≥500 (50.9%) and 62.8% had a Nadir CD4 <200. The median (IQR) duration of HIV infection was 108 months (68.9-141.4). Fourteen percent (14.6%) were on stage C at ART initiation. Concerning ART treatment, 68.2% had RAL, AZT, DDI, DAT, DDC included in their initial treatment whereas only 26.7% had those molecules in their current treatment. Few patients reported substance use (<8%, except 17.7% for tobacco (current/previous)).

In terms of the ADL and IADL instruments, 96.4% and 99.1% of the patients obtained the maximum score (6 or 4, respectively). Overall, 43.8% reported being inactive and 45.6% reported limited to moderate intensity in their physical activity.

Prevalence of physical function impairment

Almost one third of the patients had lower SPPB performance (28.8%; 95% Confident Interval (IC): 24.0-33.6) (Table 2). The SPPB mean score was 10.2±1.8. Among the components of the SPPB, the 5STS was the most altered test (64.2% [59.0-69.3] had a score of less than 4; mean score: 2.9±1.1) whereas less than 27% had lower performance for balance (mean score: 3.7±0.7) or walking speed (mean score: 3.6±0.7). The mean time to

perform the 5STS was 12.5 ±3.5 seconds. The mean walking speed was 1.3 ±0.3 meter per second. Concerning unipodal balance, 38.6% had lower performance. Overall, 45.6% [40.2 - 50.9] of the patients had physical function impairment.

Table 2. Physical Function Impairment: mean scores and prevalence

Tests	Mean score	Prevalence (%) [95%CI]
SPPB (score ≤9)	10.2 ±1.8	28.8 [24.0 - 33.7]
Balance (score <4)	3.7 ±0.7	16.2 [12.2 - 20.2]
Walking speed (speed <=0.8m/s)	3.6 ±0.7	27.0 [22.2 - 31.8]
5STS (score<4)	2.9 ±1.1	64.2 [59.0 - 69.3]
Unipodal balance (Vereeck et al, 2008)	-	38.6 [33.4 - 43.8]
Physical function impairment	-	45.6 [40.2 - 50.9]

Abbreviations: CI: Confident Interval, SPPB: Short Physical Performance Battery, 5STS: Five Sit-to-Stand

Few patients reported moderate to severe difficulties in daily physical function (<4%), except for climbing stairs (14.1%) and carrying a grocery bag (8.4%). Among patients reporting those difficulties, less than 2.5% reported severe or extreme difficulties. Only 5.4% of the patients reported at least one fall in the past year.

Description of upper extremity function

For women, the mean grip strength was 24.7 ±7.7 kg with 43.2% showing a lower grip strength whereas for men, the mean grip strength was 40.8 ±12.3 kg with 42.5% having a lower grip strength.

Associations between SPPB performance and other tests

Of 96 PLHIV with low SPPB performance, 54.2% had low performance on unipodal test (p=0.001). Concerning grip strength, men with low SPPB performance had significantly

lowe[illegible]an grip strength than other men (35.7±9.1 kg vs 41.9±12.7, p=0.005) whereas no significant difference was observed between both women's groups (p=0.93).

SPPB performance and daily physical activities

A significant association was observed between lower SPPB performance and physical inactivity (57.3%, p=0.004). Lower SPPB performance was also associated to limited to moderate intense activities (61.5%, p=0.001). Concerning daily physical activities, lower SPPB performance was also associated with difficulties in climbing stairs (45.8%) and walking one block (22.1%) (p<0.0001 for both).

Factors associated with lower SPPB performance

In univariable models, age ≥60 years (OR=2.7; CI95%=1.6-4.5), being a female (OR=2.2; CI95%=1.3-3.7), single (OR=1.8; CI95%=1.1-3.0) and non activ[illegible]OR=2.4; CI95%=1.4-3.9) were associated with lower SPPB performance. Patients with abdominal obesity (OR=2.4; CI95%=1.5-4.1), hypertension (OR=2.2; CI95%=1.2-3.8), diabetes (OR=3.0; CI95%=1.2-8.0), a longer duration of HIV infection (OR=2.7; CI95%=1.5-5.2) and who had RAL, AZT, DDI, D4T or DDC in their initial ART (OR=1.9; CI95%=1.1-3.5) also had lower SPPB performance.

In the multivariable model, age 60 years and above (adjusted OR (aOR)=3.3; CI95%=1.9-5.9), being a female (aOR=2.1; CI95%=1.1-4.1), having an abdominal obesity (aOR=2.13; CI95%=1.17-3.95), a longer duration of HIV infection (aOR=2.8; CI95%=1.5-5.6) and RAL, AZT, DDI, D4T or DDC in their initial ART (aOR=1.8; CI95%=1.0-3.3) remained associated with lower SPPB performance.

Table 3: Factors associated with lower SPPB performance in the study population

**Variables**	**Univariable model**		**Multivariable model**	
	**OR (CI 95%)***	**p-value**	**aOR (CI 95%)***	**p-value**
**Age**		**<0.0001**		
50-59 years old	1			
≥60 years old	2.69 (1.62-4.48)		3.34 (1.93-5.88)	**<0.0001**
**Gender**		**0.004**		
Men	1			
Women	2.16 (1.28-3.69)		2.14 (1.13-4.12)	**0.02**
**Marital situation**		**0.021**		
In couple	1			
Single	1.81 (1.10-3.01)			
**Level of education**		0.280		
Primary or less	1			
Secondary or more	0.76 (0.46-1.25)			
**Professional activity**		**0.001**		
Active	1			
Non active	2.36 (1.44-3.92)			
**BMI**		0.339		
Normal / underweight	1			
Overweight/obesity	1.28 (0.77-2.1)			
**Abdominal obesity**		**0.001**		
No	1			
Yes	2.43 (1.46-4.12)		2.13 (1.17-3.95)	**0.01**
**Hypertension** (ref.: no)	2.19 (1.24-3.82)	**0.006**		
**Hyperlipidemia** (ref.: no)	1.99 (0.56-6.65)	0.265		
**Diabetes** (ref.: no)	3.06 (1.19-8.05)	**0.020**		
**B or C hepatitis** (ref.: no)	0.63 (0.09-2.86)	0.585		
**Tuberculosis** (ref.: no)	1.28 (0.70-2.29)	0.420		
**Migraine** (ref.: no)	0.74 (0.31-1.67)	0.477		
**Arthrosis** (ref.: no)	1.60 (0.82-3.1)	0.165		
**Other medical problem** (ref.: no)	1.63 (0.47-5.03)	0.411		
**History of trauma** (ref.: no)	0.36 (0.08-1.12)	0.115		
**History of neurological disease** (ref.: no)	1.60 (0.78-3.25)	0.193		
**Duration of HIV infection**				
[7.29,88.4]	1			
(88.4,131]	1.80 (0.96-3.43)	0.071	1.52 (0.77-3.03)	0.227
(131,317]	2.73 (1.47-5.18)	**0.002**	2.84 (1.47-5.62)	**0.002**
**Clinical disease stages**				
A	1			
B	1.00 (0.56-1.80)	0.997		
C	0.77 (0.33-1.75)	0.547		
**Nadir CD4**				
<200	1			
≥ 200	1.27 (0.76-2.12)	0.366		

Table 3 (continued)

**Variables**	**Univariable model** OR (CI 95%)*	**p-value**	**Multivariable model** aOR (CI 95%)*	**p-value**
**Viral load**				
Detectable	1			
Undetectable	0.77 (0.39-1.60)	0.475		
**Molecules in initial ART**				
Other	1			
RAL, AZT, DDI, D4T, DDC	1.94 (1.12-3.45)	**0.020**		
**Molecules in actual ART**				
Other	1			
RAL, AZT, DDI, D4T, DDC	1.70 (0.99-2.91)	0.052	1.83 (1.01-3.28)	**0.044**
**Observance** (no)	1.60 (0.55-5.87)	0.423		
**Alcohol consumption**	0.34 (0.08-1.03)	0.088		
**Tobacco (current/previous)**	0.54 (0.25-1.09)	0.098		

DISCUSSION

In the present study, in a large sample of PLHIV aged 50 years old and above, physical function limitation evaluated with the SPPB is observed in almost 30% of the patients. As observed in western countries, the 5STS seems to be the most altered sub-test in the SPPB. PLHIV with low SPPB performance also had low unipodal balance performance and low mean grip strength (in men only). Physical function limitation seems to be more important for PLHIV aged above 60 years old, in women, for those with abdominal obesity and longer duration of the disease. The use of certain molecul[illegible]t the initial ART is also associated to lower performance.

Recent publications in older PLHIV from western countries reported similar results for the SPPB performance. In PLHIV aged 50 years or ab[illegible]living in the United States, it was reported a similar mean score (10.3 to 10.7) [18,19], a score similar to nearly 20 year older controls [18] with a trend to lower performance than those observed in controls with similar age, anthropometric and comorbidities [19]. Another study in female PLHIV reported that 20% of them performed poorly with a median score of 11, but they discussed the possibility

of a ceiling effect in their sample [20]. In a cohort of drug users (median age: 51 years old), HIV infection was also associated with a 30% higher risk of physical function limitation [21]. Functional impairment is associated with low bone and muscle mass among persons aging with HIV-infection [22]. In PLHIV but also in the general population, lower SPPB performance have already been associated with poorer outcomes such as having additional comorbidities, subsequent disability, falls, higher mortality and hospitalization rates [13,18,21,23–26], highlighting the importance of the screening of this impairment.

The most altered component of the SPPB test was the 5STS as observed in middle-aged PLHIV in western countries [9,10] and in older HIV patients [18,19]. PLHIV needed more time to complete the test than controls [19] but less than the nearly 20 years older controls [18]. As recommended in an editorial in AIDS [27], the 5STS might be the most appropriate test to evaluate physical function in standard care of HIV-infected patients. Our results also showed that this test was the most altered, but the absence of normative data adapted to our population was a limit to go further in our investigation. The results presented here are the baseline data of a 2-year longitudinal study. Data collected during the follow-up will enable us to investigate the evolution of the 5STS and to describe the factors which best predict this decline.

Lower SPPB performance has been associated with older age. The impact of aging on physical function is well-known and linked to age-related losses in skeletal muscle mass and lower aerobic capacity [28,29]. Other studies from western and African countries reported an effect of age on physical function [9–11]. We also observed that PLHIV with an abdominal obesity had lower physical performance whereas no association was observed with BMI. The impact of nutrition and nutritional status on physical function limitation has already been observed [9,11]. People with excessive body mass may have more limited endurance-based performance [30] and also have an increased cardiovascular risks. Finally as shown by

Richert et al [9], we observed that PLHIV with longer duration of the disease are more likely to have lower physical function whereas no significant association was observed with other HIV outcomes (CD4 or viral load). Interestingly, no difference in the mean duration of the disease was observed between PLHIV under 50 years and the one aged 60 years old and above (106.6 vs 104 months). The long-term effect of the virus, inflammatory process and direct or indirect effects of ART treatment could explain this association but further investigations need to be conducted. We also observed the long-term effect of some molecules included in ART treatment, such as Zidovudine, known as having myotoxic consequences. Further studies with more detailed protocol evaluating the duration of exposition to those molecules could help to understand better the phenomenon.

Concerning grip strength, in accordance to other studies in PLHIV or in African populations [18,31–33], the mean performance wer[illegible]wer in women than the men ones (24.7 kg vs 40.8 kg). The different type of activities in which women and men are engaged could explain this observation [31,34]. In a recent US study in older PLHIV, similar grip strength as in our cohort was observed in women and men (26.5 kg and 38.2 kg respectively) [18]. Weaker grip strength has also been reported in African PLHIV in comparison to HIV-uninfected subjects [35,36]. Even if men's grip strength was associated to low SPPB performance, it was not the case for women. In the present study, due to the absence of normative data developed in SSA, we decided not to go further in interpretation of the data. As lower grip strength is associated with bad outcomes (ie mortality, longer hospitalization and physical function limitation) [37], it is important to standardize procedure [38] and to have regional specific data [39]. One study in Nigeria reported normative data for adults aged between 20 to 69 years old but with a small proportion of subjects aged ≥50 years old [31].

To our knowledge, this study represents the first opportunity to describe physical function in a large sample of older PLHIV in West Africa. However, some limitations have to be mentioned. Firstly, we observed that the PLHIV included in the study reported few difficulties activities of the daily living. The ADL and IADL are scales usually used to evaluate the autonomy and the independence of older subjects (≥65 years old). Moreover, the questions in these scales might not be completely adapted to the West African population. Adapted scales have recently been published and might be more adapted to the African context [40]. Secondly, the generalizability of our results could be limited since we have included PLHIV from urban sites, under ART since at least 6 months, without important difficulties in activities of daily living and without major neurologic complications or addictive behaviors. We may have selected the most adherent PLHIV and the most engaged in medical care. However, even in this population, poor physical functioning still occurs and should not be neglected. Interestingly, beyond the fact that our population is old, this population presented some specific characteristics particularly more women than men were included and the majority did not reported substance use. Those characteristics could influence the generalizability of our results but also the comparison with other studies. Thirdly, the absence of normative data adapted to our population was a limit to go further in some aspects of our investigation. Further studies are clearly needed to depict this. It is an important point both for research and clinical domains.

CONCLUSIONS

Hence, the prevalence of physical function limitation in older PLHIV is high despite a successful antiretroviral therapy. As the 5STS was the most altered test, it could be interesting to perform it in standard care as recommended in western countries. Efforts are now needed to develop normative data in SSA populations. Lower performance seems to have consequences

on some daily activities and might be associated to higher risk of falls and fractures. Further studies are needed to describe this in more details. Longitudinal studies should also be performed to assess the evolution of the physical function limitation and their predictors. Finally, age and cardiovascular risks are main factors associated with lower physical performance. Practitioners need to promote the practice of physical activity and to educate their patients about better diet to limit the occurrence of overweight and obesity in order to prevent disability in older PLHIV. How to integrate the measurement and the management of physical function limitation in the standard of care should be investigated.

REFERENCES

1. UNIAIDS, http://aidsinfo.unaids.org/. 2016

2. Bendavid E, Ford N, Mills EJ. HIV and Africa's elderly: the problems and possibilities. AIDS Lond Engl. 2012;26 Suppl 1: S85-91. doi:10.1097/QAD.0b013e3283558513

3. Schouten J, Wit FW, Stolte IG, Kootstra NA, van der Valk M, Geerlings SE, et al. Cross-sectional Comparison of the Prevalence of Age-Associated Comorbidities and Their Risk Factors Between HIV-Infected and Uninfected Individuals: The AGEhIV Cohort Study. Clin Infect Dis Off Publ Infect Dis Soc Am. 2014;59: 1787–1797. doi:10.1093/cid/ciu701

4. Guralnik JM, LaCroix AZ, Abbott RD, Berkman LF, Satterfield S, Evans DA, et al. Maintaining mobility in late life. I. Demographic characteristics and chronic conditions. Am J Epidemiol. 1993;137: 845–857.

5. Guralnik JM, Ferrucci L, Simonsick EM, Salive ME, Wallace RB. Lower-extremity function in persons over the age of 70 years as a predictor of subsequent disability. N Engl J Med. 1995;332: 556–561. doi:10.1056/NEJM199503023320902

6. Penninx BW, Ferrucci L, Leveille SG, Rantanen T, Pahor M, Guralnik JM. Lower extremity performance in nondisabled older persons as a predictor of subsequent hospitalization. J Gerontol A Biol Sci Med Sci. 2000;55: M691-697.

7. Rusch M, Nixon S, Schilder A, Braitstein P, Chan K, Hogg RS. Impairments, activity limitations and participation restrictions: prevalence and associations among persons living with HIV/AIDS in British Columbia. Health Qual Life Outcomes. 2004;2: 46. doi:10.1186/1477-7525-2-46

8. Greene M, Covinsky KE, Valcour V, Miao Y, Madamba J, Lampiris H, et al. Geriatric Syndromes in Older HIV-Infected Adults. J Acquir Immune Defic Syndr. 2015;69: 161–167. doi:10.1097/QAI.0000000000000556

9. Richert L, Dehail P, Mercié P, Dauchy F-A, Bruyand M, Greib C, et al. High frequency of poor locomotor performance in HIV-infected patients. AIDS Lond Engl. 2011;25: 797–805. doi:10.1097/QAD.0b013e3283455dff

10. Richert L, Brault M, Mercié P, Dauchy F-A, Bruyand M, Greib C, et al. Decline in locomotor functions over time in HIV-infected patients. AIDS Lond Engl. 2014;28: 1441–1449. doi:10.1097/QAD.0000000000000246

11. Tanon A, Diallo Z, Lenaud S, Niangoran S, Peres K, Eholie S, et al. Aging with HIV Infection and Locomotors Disorders: Experience of the Infectious and Tropical Diseases Unit, Abidjan, Côte d'Ivoire. J AIDS Clin Res. 2017;08. doi:10.4172/2155-6113.1000680

12. Egger M, Ekouevi DK, Williams C, Lyamuya RE, Mukumbi H, Braitstein P, et al. Cohort Profile: the international epidemiological databases to evaluate AIDS (IeDEA) in sub-Saharan Africa. Int J Epidemiol. 2012;41: 1256–1264. doi:10.1093/ije/dyr080

13. Guralnik JM, Simonsick EM, Ferrucci L, Glynn RJ, Berkman LF, Blazer DG, et al. A short physical performance battery assessing lower extremity function: association with self-reported disability and prediction of mortality and nursing home admission. J Gerontol. 1994;49: M85-94.

14. Vereeck L, Wuyts F, Truijen S, Van de Heyning P. Clinical assessment of balance: Normative data, and gender and age effects. Int J Audiol. 2008;47: 67–75. doi:10.1080/14992020701689688

15. Katz S, Ford AB, Moskowitz RW, Jackson BA, Jaffe MW. STUDIES OF ILLNESS IN THE AGED. THE INDEX OF ADL: A STANDARDIZED MEASURE OF BIOLOGICAL AND PSYCHOSOCIAL FUNCTION. JAMA. 1963;185: 914–919.

16. Lawton MP, Brody EM. Assessment of older people: self-maintaining and instrumental activities of daily living. The Gerontologist. 1969;9: 179–186.

17. Armstrong T, Bull F. Development of the World Health Organization Global Physical Activity Questionnaire (GPAQ). J Public Health. 2006;14: 66–70. doi:10.1007/s10389-006-0024-x

18. Crane HM, Miller ME, Pierce J, Willig AL, Case ML, Wilkin AM, et al. Physical Functioning Among Patients Aging With Human Immunodeficiency Virus (HIV) Versus HIV Uninfected: Feasibility of Using the Short Physical Performance Battery in Clinical Care of People Living With HIV Aged 50 or Older. Open Forum Infect Dis. 2019;6. doi:10.1093/ofid/ofz038

19. Wallet MA, Buford TW, Joseph A-M, Sankuratri M, Leeuwenburgh C, Pahor M, et al. Increased inflammation but similar physical composition and function in older-aged, HIV-1 infected subjects. BMC Immunol. 2015;16. doi:10.1186/s12865-015-0106-z

20. Baranoski AS, Harris A, Michaels D, Miciek R, Storer T, Sebastiani P, et al. Relationship Between Poor Physical Function, Inflammatory Markers, and Comorbidities in HIV-Infected Women on Antiretroviral Therapy. J Womens Health. 2014;23: 69–76. doi:10.1089/jwh.2013.4367

21. Greene M, Covinsky K, Astemborski J, Piggott DA, Brown T, Leng S, et al. The relationship of physical performance with HIV disease and mortality: AIDS. 2014;28: 2711–2719. doi:10.1097/QAD.0000000000000507

22. Erlandson KM, Allshouse AA, Jankowski CM, MaWhinney S, Kohrt WM, Campbell TB. Functional Impairment Is Associated With Low Bone and Muscle Mass Among Persons Aging With HIV Infection: JAIDS J Acquir Immune Defic Syndr. 2013;63: 209–215. doi:10.1097/QAI.0b013e318289bb7e

23. Erlandson KM, Allshouse AA, Jankowski CM, Duong S, MaWhinney S, Kohrt WM, et al. Risk Factors for Falls in HIV-Infected Persons: JAIDS J Acquir Immune Defic Syndr. 2012;61: 484–489. doi:10.1097/QAI.0b013e3182716e38

24. Pavasini R, Guralnik J, Brown JC, di Bari M, Cesari M, Landi F, et al. Short Physical Performance Battery and all-cause mortality: systematic review and meta-analysis. BMC Med. 2016;14. doi:10.1186/s12916-016-0763-7

25. Negm AM, Kennedy CC, Pritchard JM, Ioannidis G, Vastis V, Marr S, et al. The Short Performance Physical Battery Is Associated with One-Year Emergency Department Visits and Hospitalization. Can J Aging Rev Can Vieil. 2019; 1–5. doi:10.1017/S0714980819000011

26. Lauretani F, Ticinesi A, Gionti L, Prati B, Nouvenne A, Tana C, et al. Short-Physical Performance Battery (SPPB) score is associated with falls in older outpatients. Aging Clin Exp Res. 2018 [cited 26 Jun 2019]. doi:10.1007/s40520-018-1082-y

27. Dorfman D, Saag MS. Decline in locomotor functions over time in HIV-infected patients. AIDS Lond Engl. 2014;28: 1531–1532. doi:10.1097/QAD.0000000000000302

28. Layne AS, Hsu F-C, Blair SN, Chen S-H, Dungan J, Fielding RA, et al. Predictors of Change in Physical Function in Older Adults in Response to Long-Term, Structured Physical Activity: The LIFE Study. Arch Phys Med Rehabil. 2017;98: 11-24.e3. doi:10.1016/j.apmr.2016.07.019

29. Goins RT, Innes K, Dong L. Lower Body Functioning Prevalence and Correlates in Older American Indians in a Southeastern Tribe: The Native Elder Care Study. J Am Geriatr Soc. 2012;60: 577–582. doi:10.1111/j.1532-5415.2011.03869.x

30. Manini TM, Newman AB, Fielding R, Blair SN, Perri MG, Anton SD, et al. Effects of Exercise on Mobility in Obese and Nonobese Older Adults. Obesity. 2010;18: 1168–1175. doi:10.1038/oby.2009.317

31. Adedoyin RA, Ogundapo FA, Mbada CE, Adekanla BA, Johnson OE, Onigbinde TA, et al. Reference Values for Handgrip Strength Among Healthy Adults in Nigeria. Hong Kong Physiother J. 2009;27: 21–29. doi:10.1016/S1013-7025(10)70005-1

32. Koopman JJE, van Bodegom D, van Heemst D, Westendorp RGJ. Handgrip strength, ageing and mortality in rural Africa. Age Ageing. 2015;44: 465–470. doi:10.1093/ageing/afu165

33. Ramlagan S, Peltzer K, Phaswana-Mafuya N. Hand grip strength and associated factors in non-institutionalised men and women 50 years and older in South Africa. BMC Res Notes. 2014;7: 8. doi:10.1186/1756-0500-7-8

34. Robergs R, Roberts S. Exercise Physiology: Exercise Performance and Clinical Application. St. Louis, MO: Mosby,.

35. Negin J, Martiniuk A, Cumming RG, Naidoo N, Phaswana-Mafuya N, Madurai L, et al. Prevalence of HIV and chronic comorbidities among older adults. AIDS Lond Engl. 2012;26 Suppl 1: S55-63. doi:10.1097/QAD.0b013e3283558459

36. Olsen MF, Kæstel P, Tesfaye M, Abdissa A, Yilma D, Girma T, et al. Physical activity and capacity at initiation of antiretroviral treatment in HIV patients in Ethiopia. Epidemiol Infect. 2015;143: 1048–1058. doi:10.1017/S0950268814001502

37. Bohannon RW. Muscle strength: clinical and prognostic value of hand-grip dynamometry. Curr Opin Clin Nutr Metab Care. 2015;18: 465–470. doi:10.1097/MCO.0000000000000202

38. Roberts HC, Denison HJ, Martin HJ, Patel HP, Syddall H, Cooper C, et al. A review of the measurement of grip strength in clinical and epidemiological studies: towards a standardised approach. Age Ageing. 2011;40: 423–429. doi:10.1093/ageing/afr051

39. Dodds RM, Syddall HE, Cooper R, Kuh D, Cooper C, Sayer AA. Global variation in grip strength: a systematic review and meta-analysis of normative data. Age Ageing. 2016;45: 209–216. doi:10.1093/ageing/afv192

40. Edjolo A, Pérès K, Guerchet M, Pilleron S, Ndamba-Bandzouzi B, Mbelesso P, et al. Development of the Central Africa Daily Functioning Interference Scale for Dementia Diagnosis in Older Adults: The EPIDEMCA Study. Dement Geriatr Cogn Disord. 2019;47: 29–41. doi:10.1159/000492782
